# Seroprevalence and genotypes of *Toxoplasma gondii* isolated from pigs intended for human consumption in Liaoning province, northeastern China

**DOI:** 10.1186/s13071-016-1525-2

**Published:** 2016-04-29

**Authors:** Dawei Wang, Yan Liu, Tiantian Jiang, Guoxin Zhang, Gaoming Yuan, Jianbin He, Chunlei Su, Na Yang

**Affiliations:** Laboratory of Zoonosis of Liaoning Province, College of Animal Science & Veterinary Medicine, Shenyang Agricultural University, Shenyang, Liaoning Province 110866 P.R.China; Institute of Animal Science, Chinese Academy of Agricultural Sciences, Beijing, 100193 P.R.China; Department of Microbiology, The University of Tennessee, Knoxville, Tennessee 37996 USA

**Keywords:** *Toxoplasma gondii*, Seroprevalence, Genotype, Pig, China

## Abstract

**Background:**

Genetic information for *Toxoplasma gondii* isolates from pigs in eastern, south, and southwestern regions of China has been reported previously. However, there are no data from pigs in the northeastern area of the country. To better understand the epidemiology of *T. gondii*, we determined the seroprevalence and genotypes of *T. gondii* from pigs slaughtered for human consumption in Liaoning province, northeastern China.

**Findings:**

Out of 2063 pigs examined, 233 (11.26 %) were seropositive for *T. gondii* by the modified agglutination test (MAT), and viable parasites were isolated by bioassay in mice from 23 (9.87 %) of the 233 seropositive pigs. Fifteen out of 23 isolates were genotyped using 10 PCR-restriction fragment length polymorphism (RFLP) genetic markers including SAG1, SAG2, SAG3, BTUB, GRA6, c22-8, c29-2, L358, PK1 and Apico. One isolate was identified as ToxoDB genotype #3 (type II-variant), and one was genotype #1 or #3. The other 13 isolates were ToxoDB #9 (type Chinese 1).

**Conclusions:**

To our knowledge, this is the first report of *T. gondii* isolation and genotyping from pigs in northeastern China. This study indicates that pigs are a potential source for transmission of *T. gondii* to humans, therefore poses a potential public health concern. The genotyping results revealed the presence of genotype Chinese 1 in northeastern China, enriching the scope of *T. gondii* genotypes distribution in eastern Asia.

## Introduction

*Toxoplasma gondii* is an obligate intracellular zoonotic protozoan, infecting warm-blooded animals, including humans. An estimated one-third of the human population worldwide and 7.9 % of the population in China are chronically infected with *T. gondii* [1–4]. In women, primary infection during pregnancy can cause severe damage to fetus and newborns including blindness, abortion and stillbirth. It can cause severe infections in individuals with compromised immune systems. Humans can acquire infection via three major ways, i.e. consumption of undercooked meat containing *T. gondii* tissue cysts, inadvertently ingesting oocysts in water, soil, vegetables and fruits, and transplacental transmission [5].

Given the biological and epidemiological diversity of *T. gondii*, it is expected that the parasite is genetically diverse. The distribution of *T. gondii* genotypes varies in different geographic regions. Early studies have shown that *T. gondii* isolates from Europe and North America were grouped into three main clonal lineages, i.e. Types I, II and III. Type I is generally more virulent than types II and III [6]. However, recent research data revealed that there is limited diversity and only a few genotypes of *T. gondii* are prevalent in Europe, North America, Africa and Asia. In contrast, a large number of highly diverse genotypes exists in Central and South America [7]. In China, the genotype #9 (Chinese 1) is widespread and is considered the main genotype [8–12].

Pigs are an important source of *T. gondii* infection in human populations [1]. Pork is the main meat source for human consumption in Liaoning Province. In China, although there are some studies concerning the genetic information for *T. gondii* isolates in pigs from Guangdong, Yunnan, Anhui, Guizhou, Jiangxi, Sichuan and Chongqing [8, 12–16], there are no data from pigs in the northeastern region of the country. In the present study, we report the prevalence and genetic characteristics of *T. gondii* isolates from pigs intended for human consumption in Liaoning Province, northeastern China.

## Methods

### Ethics statement

All animals were handled in strict accordance with good animal practice according to the Animal Ethics Procedures and Guidelines of the People’s Republic of China, and the study was approved by the Animal Ethics Committee of Shenyang Agricultural University (Permit No. SYXK<Liao>2011-0001).

### Sample collection

Between April 2013 and December 2014, hearts of 2063 pigs slaughtered for human consumption were obtained for the present study from five slaughterhouses in Liaoning province, northeastern China. These pigs were from Shenyang, Tieling, Kaiyuan, Xinmin and Faku. Heart fluid was centrifuged to collect supernatant for serology test of *T. gondii* infection.

### Detection of *T. gondii* antibodies by MAT

Sera of pigs were tested for the specific antibodies to *T. gondii* by the modified agglutination test (MAT) as described by Dubey & Desmonts [17]. Two-fold dilutions of sera were performed from 1:25 to 1:3200. The test was considered positive when a layer of agglutinated parasites was formed in wells at dilutions of 1:25 or higher; positive and negative controls were included in each test.

### Bioassay in mice

Heart tissues from seropositive pigs were used to isolate *T. gondii* by bioassay in Kunming (KM) mice following the standard protocol [18]. The homogenate of pig heart tissue was inoculated (1 ml/mouse) s.c. into five female KM mice. Mice were observed on a daily basis. After 60 days mice were killed, their brain tissue was obtained for tissue cysts examination. The brain of each mouse was homogenated according to the above-mentioned procedures. Each homogenate was inoculated (1 ml/mouse) i.p. into five female KM mice. Then, 1 mg/Kg dexamethasone was injected into each mouse in the first 3 days after inoculation of homogenate. The isolates were collected from the peritoneal fluids of mice infected with *T. gondii* for 7–14 days.

### Genotying of *T. gondii* isolates from pigs

The genomic DNA of *T. gondii* was extracted from peritoneal fluids of infected mice using DNA Isolation Kit (Invitrogen). Genotyping was carried out for the 10 PCR-restriction fragment length polymorphism (RFLP) genetic markers including SAG1, SAG2 (5'-3'SAG2, alt.SAG2), SAG3, BTUB, GRA6, c22-8, c29-2, L358, PK1 and Apico [19].

## Results

In the present study, 233 (11.26 %) of 2063 pigs were seropositive for *T. gondii*, with titers of 1:25 in 108, 1:50 in 58, 1:100 in 14, 1:200 in 6, 1:400 in 13, 1:800 in 27, 1:1600 in 6 and 1:3200 in 1 (Table 1). Viable *T. gondii* strains were isolated in mice from 23 (9.87 %) of 233 seropositive pigs, one with titers of 1:100, eight with titers of 1:400, 13 with titers of 1:800, and one with titers of 1:1600 in this study (Table 1). The isolation results indicated that probability of viable *T. gondii* isolation was high at higher titers (1:400, 1:800). The designation of those isolates is shown in Table 2.

**Table 1:** Serological and parasitic prevalence of *T. gondii* infection in 2063 pigs from Liaoning province, northeastern China

MAT titers^a^	No. of positive pigs	No. of *T. gondii* isolates (%)
1:25	108	0 (0)
1:50	58	0 (0)
1:100	14	1 (7.14)
1:200	6	0 (0)
1:400	13	8 (61.54)
1:800	27	13 (48.15)
1:1600	6	1 (16.67)
1:3200	1	0 (0)
Total	233	23 (9.87)

^a^MAT, modified agglutination test

**Table 2:** Isolation of *T. gondii* from hearts of pigs from slaughterhouses in Liaoning Province, northeastern China

Pig No.	Date	MAT titer	Isolation designation
3-2	5/1/2013	100	TgPigCn3-2
3-12	5/1/2013	800	TgPigCn3-12
5-5	5/14/2013	800	TgPigCn5-5
5-6	5/14/2013	400	TgPigCn5-6
5-7	5/14/2013	400	TgPigCn5-7
8-16	5/21/2013	800	TgPigCn8-16
8-18	5/21/2013	400	TgPigCn8-18
10-34	5/31/2013	800	TgPigCn10-34
14-56	6/18/2013	400	TgPigCn14-56
19-24	11/13/2013	800	TgPigCn19-24
22-10	11/20/2013	800	TgPigCn22-10
27-8	12/22/2013	800	TgPigCn27-8
29-5	12/30/2013	800	TgPigCn29-5
33-6	3/13/2014	800	TgPigCn33-6
40-10	3/28/2014	1600	TgPigCn40-10
58-14	8/12/2014	800	TgPigCn58-14
61-11	8/14/2014	800	TgPigCn61-11
69-2	11/3/2014	400	TgPigCn69-2
69-14	11/3/2014	400	TgPigCn69-14
77-2	11/24/2014	800	TgPigCn77-2
80-11	12/7/2014	800	TgPigCn80-11
80-25	12/7/2014	400	TgPigCn80-25
80-27	12/7/2014	400	TgPigCn80-27

Due to low DNA concentration, eight of 23 isolates could not be genotyped with complete data at all loci, including TgPigCn3-2, TgPigCn5-7, TgPigCn22-10, TgPigCn69-2, TgPigCn69-14, TgPigCn77-2, TgPigCn80-25 and TgPigCn80-27, and therefore data from these eight strains were not included. The results of genotyping of these 15 strains and eight references were summarized in Table 3. TgPigCn5-6 was identified as ToxoDB#3 (type II-variant), TgPigCn14-56 had type II alleles at all markers except one allele not identified at the Apico locus and are considered the ToxoDB #1 or #3, and the other 13 isolates were ToxoDB #9 (type Chinese 1), which suggested that ToxoDB #9 is predominant in this region.

**Table 3:** Genotyping of *T. gondii* from pigs in northeastern China

Isolate ID	SAG1	5'-3'SAG2	alt-SAG2	SAG3	BTUB	GRA6	c22-8	C29-2	L358	PK1	Apico	Genotype
GT1	I	I	I	I	I	I	I	I	I	I	I	Reference,ToxoDB#10, type I
PTG	II or III	II	II	II	II	II	II	II	II	II	II	Reference,ToxoDB #1, type II
CTG	II or III	III	III	III	III	III	III	III	III	III	III	Reference,ToxoDB#2, type III
Coug	I	II	II	III	II	II	II	u-1	I	u-2	I	Reference, ToxoDB#66
MAS	u-1	I	II	III	III	III	u-1	I	I	III	I	Reference, ToxoDB#17
TgCatBr5	I	III	III	III	III	III	I	I	I	u-1	I	Reference, ToxoDB#19
TgCatBr64	I	I	u-1	III	III	III	u-1	I	III	III	I	Reference, ToxoDB#111
TgRsCr1	u-1	I	II	III	I	III	u-2	I	I	III	I	Reference, ToxoDB#52
This study												
TgPigCn3-12	u-1	II	II	III	III	II	II	III	II	II	I	ToxoDB#9, Chinese 1
TgPigCn5-5	u-1	II	II	III	III	II	II	III	II	II	nd	ToxoDB#9, Chinese 1
TgPigCn5-6	II or III	II	II	II	II	II	II	II	II	II	I	ToxoDB#3, type II-variant
TgPigCn8-16	u-1	II	II	III	III	II	II	III	II	II	nd	ToxoDB#9, Chinese 1
TgPigCn8-18	u-1	II	II	III	III	II	II	III	II	II	I	ToxoDB#9, Chinese 1
TgPigCn10-34	u-1	II	II	III	III	II	II	III	II	II	nd	ToxoDB#9, Chinese 1
TgPigCn14-56	II or III	II	II	II	II	II	II	II	II	II	nd	ToxoDB#1 or #3
TgPigCn19-24	u-1	II	II	III	III	II	II	III	II	II	I	ToxoDB#9, Chinese 1
TgPigCn27-8	u-1	II	II	III	III	II	II	III	II	II	I	ToxoDB#9, Chinese 1
TgPigCn29-5	u-1	II	II	III	III	II	II	III	II	II	I	ToxoDB#9, Chinese 1
TgPigCn33-6	u-1	II	II	III	III	II	II	III	II	II	I	ToxoDB#9, Chinese 1
TgPigCn40-10	u-1	II	II	III	III	II	II	III	II	II	I	ToxoDB#9, Chinese 1
TgPigCn58-14	u-1	II	II	III	III	II	II	III	II	II	I	ToxoDB#9, Chinese 1
TgPigCn61-11	u-1	II	II	III	III	II	II	III	II	II	nd	ToxoDB#9, Chinese 1
TgPigCn80-11	u-1	II	II	III	III	II	II	III	II	II	nd	ToxoDB#9, Chinese 1

## Discussion

Seroprevalence of *T. gondii* in pigs varies in different reports from China. In the present study, we found a prevalence of 11.26 % (233/2063) in Liaoning Province. Other studies reported 70.7 % in Guizhou Province [8], 10.1 % in Anhui Province [15], and 22.3 % in Yunnan Province [20]. Taken together, pork may pose a potential risk of *T. gondii* infection in humans in China.

Genotype #9 has been identified in a variety of domestic and wild animals in China. Here we summarized genotyping data in China in Table 4. Genotype #9 was previously identified in 38 pigs including two in Guangdong, one in Gansu, six in Henan, five in Yunnan, one in Anhui, seven in Guizhou, three in Sichuan, one in Chongqing and 12 in Jiangxi provinces [8, 11–16]. Genotype #9 was also reported in 92 cats including 11 in Beijing, 40 in Guangdong, nine in Anhui, two in Shanxi, seven in Guizhou, six in Hubei, 11 in Yunnan, one in Henan and five in Zhejiang provinces [8–12, 21–24]. The same genotype was also identified in one black goat in Yunnan Province [25], one vole in Hubei Province [12], five bats in Guangdong, Jiangxi and Jilin provinces [4], seven rats and mice in Jiangsu Province [26], and 12 humans in Anhui, Zhejiang and Guangdong provinces [10, 11, 27]. The compiled results indicated that ToxoDB #9 is the most prevalent genotype in different hosts and geographical locations in Mainland China. The same genotype was also identified in dogs from Sri Lanka and Colombia, in chickens from Brazil and in sheep from the United States, indicating that this genotype is widespread worldwide [12, 27].

**Table 4:** Genotyping of *T. gondii* in animals and humans by PCR-RFLP in different geographic regions of China

Host	Location	Samples	ToxoDB PCR-RFLP genotypes												Reference
			#10	#1	#2	#3	#9		# 17	# 20	# 204	# 205	# 213	# 225	
			Type I	Type II	Type III	Type II-variant	Chinese 1	Chinese 2							
Cat	BGAGHYSHZ^a^	109^e^	1	2	1	1	92	2^i^	1	1		4		2	[8–12, 21–24]
Pig	GHQNGYAGJSCHHJL^b^	71	15	1^f^		2	51						2		[8, 11–16, 33]; this study
Sheep	Qinghai	1				1									[11]
Goat	Yunnan	8	7				1								[25]
Rabbit	Shanghai	1			1										[31]
Wild bird	Xinjiang	3	1			2									[28]
Pet bird	Gansu	4				4									[29]
Chicken	Anhui	1												1	[12]
Sparrow	Gansu	4^g^				3									[30]
Voles	Hubei	1					1								[12]
Bats	JGJ^c^	8	3				5								[4]
Rat/mouse	Jiangsu	7					7								[26]
Dog	Henan	1^h^													[32]
Human	AGZS^d^	16	2	1			12				1				[10, 11, 27]
Total		235	29	4	2	13	169	2	1	1	1	4	2	3	

^a^BGAGSHYS, Beijing, Guangdong, Anhui, Guizhou, Hubei, Yunnan, Shanxi, Henan, Zhejiang

^b^GHQNGYAGJSCHHJL, Guangdong, Hunan, Qinghai, Nanjing, Gansu, Yunnan, Anhui, Guizhou, Jiangxi, Sichuan, Chongqing, Hubei, Henan, Jilin, Liaoning

^c^JGJ, Jilin, Guangdong, Jiangxi

^d^AGZS, Anhui, Guangdong, Zhejiang, Shanghai

^e^Two of 109 samples was identified as a new genotype and not listed in Table 4

^f^TgPigCn14-56 had type II alleles at all markers except a allele not identified at the Apico locus and are considered the ToxoDB #1 or #3 in this study

^g^One of four was identified as a new genotype and not listed in Table 4

^h^This genotype was identified as a new genotype and not listed in Table 4

^i^Genotype Chinese 2 was identified in two cats in this study [9]

In this study, ToxoDB#3 (the type II-variant) was identified in one pig from Liaoning Province. This genotype was also identified in one cat in Yunnan Province [23], in one pig in Guangdong Province [16], in one sheep from Qinghai Province [11], in two wild birds in Xinjiang [28], in four pet birds from Gansu [29], in three sparrows from Gansu [30] (Table 4). TgPigCn14-56 was not successfully typed at locus Apico; however, based on the genotype profile of the other nine alleles it is either ToxoDB genotye #1 (type II) or #3 (type II variant).

## Conclusions

In conclusion, the present study is the first report of *T. gondii* isolates and genotyping from pigs in Liaoning Province, northeastern China, and the results extended the scope of *T. gondii* genotype database in China. The results indicated that *T. gondii* infection was widespread in pigs intended for human consumption in this region, which may serve as an important source for transmission of the parasite and poses a public health concern.
